# Dynamic Dissection of Dynein and Kinesin-1 Cooperatively Mediated Intercellular Transport of Porcine Epidemic Diarrhea Coronavirus along Microtubule Using Single Virus Tracking

**DOI:** 10.1080/21505594.2021.1878748

**Published:** 2021-02-04

**Authors:** Wei Hou, Wenjie Kang, Yangyang Li, Yanke Shan, Shouyu Wang, Fei Liu

**Affiliations:** a Joint International Research Laboratory of Animal Health and Food Safety of Ministry of Education & Single Molecule Nanometry Laboratory (Sinmolab), Nanjing Agricultural University, Nanjing, Jiangsu, China; b Computational Optics Laboratory, Jiangnan University, Wuxi, Jiangsu, China

**Keywords:** Dynein, intracellular transport, kinesin-1, microtubule, porcine epidemic diarrhea virus (PEDV)

## Abstract

It is now clear that the intercellular transport on microtubules by dynein and kinesin-1 motors has an important role in the replication and spread of many viruses. Porcine epidemic diarrhea virus (PEDV) is an enveloped, single-stranded RNA virus of the Coronavirus family, which can infect swine of all ages and cause severe economic losses in the swine industry. Elucidating the molecular mechanisms of the intercellular transport of PEDV through microtubule, dynein and kinesin-1 will be crucial for understanding its pathogenesis. Here, we demonstrate that microtubule, dynein, and kinesin-1 are involved in PEDV infection and can influence PEDV fusion and accumulation in the perinuclear region but cannot affect PEDV attachment or internalization. Furthermore, we adopted a single-virus tracking technique to dynamically observe PEDV intracellular transport with five different types: unidirectional movement toward microtubule plus ends; unidirectional movement toward microtubule minus ends; bidirectional movement along the same microtubule; bidirectional movement along different microtubules and motionless state. Among these types, the functions of dynein and kinesin-1 in PEDV intercellular transport were further analyzed by single-virus tracking and found that dynein and kinesin-1 mainly transport PEDV to the minus and plus ends of the microtubules, respectively; meanwhile, they also can transport PEDV to the opposite ends of the microtubules different from their conventional transport directions and also coordinate the bidirectional movement of PEDV along the same or different microtubules through their cooperation. These results provided deep insights and references to understand the pathogenesis of PEDV as well as to develop vaccines and treatments.

## Introduction

PEDV is a member of the Alphacoronavirus family and shares the common features of the genomic organization, replication strategy, and the function of a part of the viral non-structural proteins with other Coronaviruses [1,2]. PEDV can infect swine of all ages and cause watery diarrhea, vomiting, and dehydration. PEDV was first reported in 1971 in the UK [3], and afterward was discovered in Europe and Asia [4–7]. In 2013, the PEDV epidemic wave first occurred in the USA, and over 2000 cases were registered, mainly in high-density pig farms with high morbidity and mortality (~100%) in piglets [8]. Though the US Department of Agriculture (USDA) conditionally licensed vaccines against PEDV, the PEDV outbreaks continue and have now spread to Mexico, Peru, Canada, and other countries; and in particular, the repeated outbreaks occurred in the same farms that were previously infected with PEDV. These PEDV outbreaks caused severe economic losses in the swine industry.

To understand PEDV outbreaks, various studies have been reported especially on the receptors [9,10] and endocytic pathways [11,12]. However, little is known about how PEDV particles traverse across the cytoplasm to facilitate their infection. It is known that the cytoplasm of a cell has high density with organelles, proteins, and RNAs, which restrict random diffusional movements of the virus to its required destinations within the cell. Crowding with macromolecules makes it necessary for active transport along microtubules by molecular motor proteins, specifically dynein and kinesins [13–15]. In many cell types, cytoplasmic dynein motors transport cargoes in a retrograde manner toward the minus end of microtubules, which are frequently anchored at the microtubule-organizing center (MTOC) [16,17]. Kinesin, on the other hand, transports cargoes mainly in an anterograde manner toward the plus end of microtubules, which are often located in the cell periphery [18,19]. These two motors are required for many functions in cells, including mRNA transport, vesicular trafficking, endoplasmic reticulum positioning, transport of flagellar components, movement of signaling proteins, as well as spindle microtubule and chromosomal movements [20,21]. Interestingly, many viruses are capable of subverting the microtubule transport system to facilitate their replication: viruses such as human immunodeficiency virus (HIV) [22] and parvoviruses [23] require microtubules and microtubule-associated motor proteins dynein and kinesin during cell entry for efficient nuclear targeting, either for cytosolic transport of naked viral particles or for transport inside vesicles. *In vitro* motility assays clearly indicate that cytoplasmic dynein and kinesin can function independently to produce motility in opposite directions along microtubules, but multiple studies have also suggested that the activities of these two motors are coordinately coupled in the cell [24]; thus, these two motors may lead to the complex movements along microtubules in live cells [25–28]. Moreover, as a highly dynamic process, intracellular transport can hardly be truly reflected in fixed cell assays via traditional molecular biological methods. Both the complex movements and the highly dynamic process of the intercellular transport limit the knowledge of the mechanism on the interaction between PEDV particles and host cells, thereby hindering the effective prevention and treatment of PEDV.

In order to unveil the role of microtubule, dynein and kinesin-1 in PEDV infection, we first determined that microtubule, dynein and kinesin-1 are involved in PEDV infection, and can influence PEDV fusion and accumulation in the perinuclear region but cannot affect PEDV attachment or internalization. Then, we adopted a single-virus tracking technique to observe transient and individual events on PEDV intracellular transport in live cells at a single-virus level. It is the first time that five types of PEDV intracellular transport along microtubules driven by dynein and kinesin-1 were dynamically observed. Among these types, the functions of dynein and kinesin-1 in PEDV intercellular transport were further analyzed by single-virus tracking and found that dynein and kinesin-1 mainly transport PEDV to the minus and plus ends of the microtubules, respectively. Meanwhile, dynein and kinesin-1 also can transport PEDV to the opposite ends of the microtubules different from their conventional transport directions and also coordinate the bidirectional movement of PEDV along the same or different microtubules through their cooperation. These results were providing references for understanding the pathogenesis of PEDV and other Coronaviruses as well as for vaccine and drug development.

## Materials and methods Virus production, purification and labeling

First, Vero (African green monkey kidney) cells were grown to a monolayer in Dulbecco’s modified Eagle’s medium (DMEM, Gibco, USA) supplemented with 10% fetal bovine serum (FBS, Gibco, USA) and maintained at 37°C with 5% CO_2_. Then, the cells were washed three times with serum-free DMEM, and cultured with 0.3% tryptose phosphate broth (TPB, Sigma, USA) and 3 μg/mL trypsin (Sigma, USA) in the DMEM. PEDV strain CV777 was propagated in this Vero cell monolayer at 37°C with 5% CO_2_ for 72 h. Afterward, the progeny virus was collected from the cells, whose debris was removed by centrifugation at 850 g for 10 min after freezing and thawing twice. Finally, the virus was purified by a 10%-60% gradient of sucrose at 100,000 g at 4°C for 2 h. For labeling the lipophilic fluorescent dye, the purified PEDV was incubated with 1,1ʹ-dioctadecyl-3,3,3ʹ,3ʹ-tetramethylindodicarbocyanine, 4-chlorobenzenesulfonate salt (DiD) (Invitrogen, USA) at room temperature for 1 h. Unbound dye was removed by gel filtration on a NAP-10 column (GE Healthcare, USA).

## Virus titer assays

To investigate the influence of DiD on PEDV infectivity, DiD-labeled and unlabeled PEDV particles were diluted by 10-fold ranging from 10^−1^ to 10^−8^ in DMEM with 0.3% TPB and 3 μg/mL trypsin and then introduced to 96-well plates in Vero cell monolayer. Next, the infected cells were cultured in an incubator at 37°C with 5% CO_2_ for 24 h, and the virus titer was finally quantified by observing and counting the number wells with cytopathic effect (CPE) on Vero cells according to the Reed and Muench method [29].

## Cell culture and drug treatment

Vero cells were grown in DMEM with 10% FBS in a 5% CO_2_ incubator. To disrupt the microtubule assembly or inhibit the dynein function, cells were incubated with a medium containing 25 μM nocodazole (Sigma-Aldrich, USA) or ciliobrevin D (Sigma-Aldrich, USA) [30] for 60 min before experiments, respectively. The drugs were maintained in the culture throughout the experiments.

## Plasmid and siRNA

The siRNA sequences targeting kinesin-1 heavy chain (KIF5B) were prepared by Sangon Biotech (China). The target DNA sequence for siRNA KIF5B is 5ʹ-CAAGCAAGACAAGACTTGAAGGGTT −3ʹ [31], and the control siRNA is 5ʹ-TTCTCCGAACGTGTCACGTTT-3ʹ. These siRNA sequences were transfected into Vero cells in a 6-well plate using PepMute siRNA Transfection Reagent (SignaGen, USA). A second transfection was performed 24 h later. After 48 h from the first transfection, cells were collected and plated in a new 6-well plate or in a 35 mm-confocal dish with a proper density, and finally transiently transfected with plasmids encoding EGFP-microtubule, mKO2-dynein or mKO2-KIF5B using LipoMax transfection reagents (Sudgen, USA). After an additional 24 h post-transfection, the cells were used for the later experiments.

## Reverse Transcriptase Real Time-PCR

To determine the influence of drug or siRNA on PEDV infection, samples at 0 h and 1 h post-infection with and without drug or siRNA were collected for RT-PCR. Total RNA was extracted from cells by using RNA-easy Isolation Reagent (Vazyme, China), and RNA was reversely transcripted using HiScript® II Q RT SuperMix for qPCR (Vazyme, China). The primer sequences used for PEDV detection are 5ʹ-GCACTTATTGGCAGGCTTTGT-3ʹ and 5ʹ-CTACGACACTTTCTTTTCTCAATGG-3ʹ. The amount of the viral RNA was quantified by AceQ qPCR SYBR Green Master Mix (Vazyme, China).

## Western blot

Cell samples were washed twice with PBS and lysed with NP-40 lysis buffer (Beyotime, China) supplemented with protease inhibitor phenylmethanesulfonylfluoride (PMSF, Beyotime, China). After centrifugation at 5652 g at 4°C for 10 min, the supernatants of the cell lysates were normalized for equal protein content using a BCA protein assay kit (GenStar, China). Equal amounts of samples were loaded into 10% polyacrylamide gel for SDS-polyacrylamide gel electrophoresis (SDS-PAGE). After separation, the protein was transferred to 0.2 μm-nitrocellulose blotting membrane (GE Healthcare, USA) and detected by incubation with monoclonal antibody against N protein of PEDV, anti-GAPDH mouse monoclonal antibody (Proteintech, USA), or anti-KIF5B rabbit polyclonal (Proteintech, USA). Finally, HRP-conjugated secondary antibody was added and treated with enhanced chemiluminescence (ECL) buffer (Vazyme, China). The bands were quantified with ImageJ software.

## Fluorescence co-localization assay

Vero cells were incubated in a 35 mm-confocal dish with a proper density and then transfected with plasmids encoding EGFP-microtubule with mKO2-KIF5B or mKO2-dynein using LipoMax transfection reagents. After 24 h, cells were infected with DiD-labeled PEDV particles at 4°C for 30 min for virus attachment, and next incubated at 37°C for 5 min. Finally, cells were fixed for observation.

## Virus fusion assay

Vero cells were treated with control DMSO, nocodazole, ciliobrevin D, control siRNA, and KIF5B siRNA, respectively. Afterward, these cells were infected with DiD-labeled PEDV particles for 1 h and then fixed by 4% paraformaldehyde. To visualize the outline of cells, F-actin close to the plasma membrane was stained with AbFluor 488-conjugated phalloidin (Abbkine, China) without permeabilization. In these conditions, some phalloidin penetrated through the cell membrane and bound the cortical actin [32]. The samples were observed using a confocal microscope, and the DiD fluorescence signal was analyzed using ImageJ.

## Live cell imaging and analysis

Vero cells were grown in a 35 mm-confocal dish and transfected with plasmids encoding EGFP-microtubule with mKO2-dynein or mKO2-KIF5B using LipoMax transfection reagents. After 24 h post-transfection, cells were infected with DiD-labeled PEDV particles at 4°C for 30 min for virus attachment, and then cultured in a microscopic incubation system (Tokai Hit, Japan) maintained at 37°C and supplied with 5% CO_2_. Finally, three-color fluorescence images were recorded with an interval of 1.5 s using a fluorescence microscope (Nikon A1 Plus si STORM).

The fluorescence intensity was analyzed using NIS-Elements software and ImageJ. First, each captured frame was processed using a Gaussian spatial filter to remove background and noise. Then, each single virion was located in each frame and its trajectory was determined from multiple frames. Finally, the virion velocity was calculated to describe the virus motions.

## Results Microtubule, dynein and kinesin-1 are involved in PEDV infection

**Figure 1.**
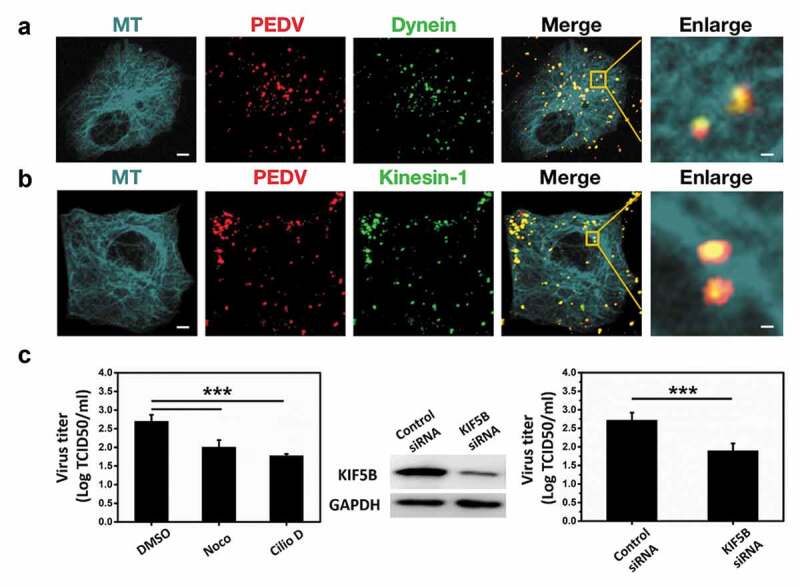
Microtubule, dynein and kinesin-1 are involved in PEDV infection in Vero cells. (a)/(b) Fluorescence images of DiD-labeled PEDV particles in Vero cells respectively transfected with EGFP-microtubule and mKO2-dynein/mKO2-KIF5B. Scale bars indicate 5 μm in the whole fields of view (FoVs) and 1 μm in the zoomed-in FoVs. (c) Titers of PEDV particles from control DMSO, nocodazole (Noco), ciliobrevin D (Cilio D), control siRNA and KIF5B siRNA pretreated Vero cells. The KIF5B level was determined by Western blot using the KIF5B antibody, and equal loading was verified with the anti-GAPDH antibody. Each data point represents mean ± standard deviation from three independent experiments. Statistical analysis on all data was performed using one-way ANOVA (***, P < 0.001)

After reaching the cytoplasm, viruses are delivered to the host cell cytoplasm from the plasma membrane to the site of viral replication, which associates with microtubule-based motors, such as dynein and kinesin. To investigate the role of microtubule, dynein, and kinesin-1 in PEDV infection, PEDV particles were labeled with the lipophilic fluorescent probe DiD, which did not significantly influence the PEDV infectivity as shown in Figure S1. Moreover, based on its hydrophobic feature [33], DiD can be inserted into the phospholipid bilayer of the virus membrane for viral labeling. After labeling, the surface density of the DiD dye was sufficiently high so that its fluorescence emission was quenched but still allowed single dye-labeled viruses to be clearly detected; therefore, most of the DiD signals represented DiD-labeled PEDV particles. Vero cells previously transfected with plasmids encoding EGFP-microtubule and mKO2-dynein or mKO2-KIF5B were infected with DiD-labeled PEDV particles. Fluorescence images shown in Figure 1(a) and 1(b) suggest that PEDV particles colocalized with microtubule and dynein or kinesin-1, thus indicating that microtubule, dynein, and kinesin-1 directly interacted with PEDV particles. To further test whether they could influence PEDV infection, Vero cells were first treated with control DMSO, 25 μM nocodazole (Noco) as a microtubule inhibitor, 25 μM ciliobrevin D (Cilio D) as a dynein inhibitor, control siRNA, and KIF5B siRNA as knockdown of KIF5B (kinesin-1 heavy chain), respectively, and then infected with PEDV particles for 4 h. Titers of PEDV particles were measured as shown in Figure 1(c), and these results show that the inhibition of microtubule and dynein as well as the knockdown of KIF5B significantly reduced the PEDV infection, proving that microtubule, dynein, and kinesin-1 were involved in PEDV infection.

## Microtubule, dynein, and kinesin-1 affect PEDV fusion and accumulation in the perinuclear region

Given microtubule, dynein and kinesin are required for intercellular transport and microtubule for efficient fusion with endosomal membrane [34], the role of microtubule, dynein, and kinesin-1 in the fusion and the transport of incoming PEDV particles to the perinuclear region was tested.

**Figure 2.**
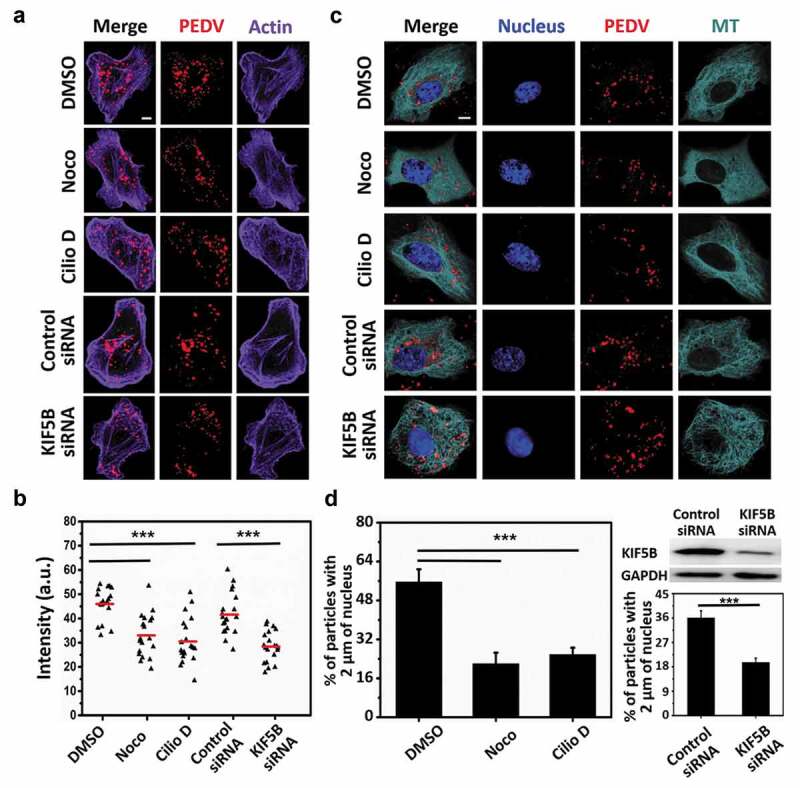
Microtubule, dynein and kinesin-1 affect PEDV fusion and accumulation in the perinuclear region. (a) Fluorescence images of Vero cells first respectively treated with control DMSO, Noco, Cilio D, control siRNA and KIF5B siRNA, then infected with DiD-labeled PEDV particles for 1 h and finally fixed. Actin was stained with AbFluor 488-conjugated phalloidin. Scale bar, 5 μm. (b) Statistical analysis on the DiD fluorescence intensity from (a) (n = 20 cells). a.u.: arbitrary units. (c) Fluorescence images of Vero cells first transfected with EGFP-MT and respectively treated with control DMSO, Noco, Cilio D, control siRNA and KIF5B siRNA and then infected with DiD-labeled PEDV particles. Nuclei were stained with DAPI. Scale bar, 5 μm. (d) Quantification on the percentage virions within 2 μm of the nuclei in 15 infected cells respectively treated with control DMSO, Cilio D, Noco, control siRNA and KIF5B siRNA. The KIF5B level was determined using Western blot with the KIF5B antibody, and equal loading was verified using the anti-GAPDH antibody. Each data point represents mean ± standard deviation from three independent experiments. Statistical analysis of all data was performed using one-way ANOVA (***, P < 0.001)

First, the fixed imaging approaches on PEDV fusion were carried out. Vero cells, respectively, treated with control DMSO, Noco, Cilio D, control siRNA, and KIF5B siRNA were infected with the same amount of DiD-labeled PEDV particles for 1 h and then fixed and stained with AbFluor 488-conjugated phalloidin as shown in Figure 2(a). The fusion was determined by quantifying the DiD fluorescence intensity in the infected Vero cells as shown in Figure 2 (b). It is because a significant increase in fluorescence intensity is expected when viruses fuse in the cells [35–37], and after fusion, the DiD signal still remains high for a period of time (up to 30 min) [36]. These results show that the DiD fluorescence intensities were lower in the Noco, Cilio D, and KIF5B siRNA treated cells compared to those in the DMSO and control siRNA treated cells, suggesting that the inhibition of microtubule and dynein as well as the knockdown of KIF5B significantly reduced the PEDV fusion.

Moreover, in order to double-check PEDV fusion, another experiment was implemented. It is known that all Coronavirus (CoV) virions contain a canonical set of four structural proteins. The viral genomic RNA is encapsidated by the nucleocapsid protein (N) to form the helical nucleocapsid, which is surrounded by the membrane glycoprotein (M), the small envelope protein (E), as well as the spike glycoprotein (S) [38]. During the infection, enveloped viruses need to fuse their envelopes with the host cell membrane to deliver nucleocapsid to the target cells [39]. Therefore, after enveloped virus fusion, its S protein and N protein will be separated. In order to determine the roles of microtubule, dynein and kinesin-1 in PEDV fusion, we performed the following procedures. Vero cells were first treated with control DMSO, Noco, Cilio D, control siRNA, and KIF5B siRNA, respectively, and then infected with the same amount of PEDV particles for 1 h and finally fixed. Fixed cells were then stained for S protein and N protein. The colocalization between PEDV S protein and N protein in Vero cells was detected as shown in Figure S2, the higher colocalization between PEDV S protein and N protein was found in the Noco, Cilio D, and KIF5B siRNA treated cells compared to those in the DMSO and control siRNA treated cells, illustrating that the inhibition of microtubule and dynein as well as the knockdown of KIF5B significantly reduced the PEDV fusion, which was consistent with the result of Figure 2(b).

Next, the fixed imaging approaches on PEDV accumulation in the perinuclear region were also implemented. Vero cells transfected with EGFP-MT and treated with control DMSO, Noco, Cilio D, control siRNA, and KIF5B siRNA were infected with DiD-labeled PEDV particles and fixed at 1 h post-infection as shown in Figure 2(c). The numbers of PEDV particles within 2 μm of the nuclei were measured as shown in Figure 2(d). The results show that more viral particles remained scattered and distanced from the nuclei in the Noco, Cilio D, and KIF5B siRNA treated cell conditions than those in the control DMSO and control siRNA treated cell conditions, suggesting that the inhibition of microtubule and dynein as well as the knockdown of KIF5B significantly reduced the PEDV accumulation in the perinuclear region.

According to Figure 2, the results illustrate that the inhibition of microtubule and dynein as well as the knockdown of KIF5B significantly reduced PEDV fusion and accumulation in the perinuclear region, proving that microtubule, dynein, and kinesin-1 are required for PEDV fusion and accumulation in the perinuclear region. However, it is also possible that the inhibition of microtubule and dynein as well as the knockdown of KIF5B may also decrease the attachment and internalization of PEDV, thus decreasing PEDV fusion and accumulation in the perinuclear region. To this problem, the influence of microtubule, dynein, and kinesin-1 on PEDV attachment and internalization should be analyzed.

## Microtubule, dynein, and kinesin-1 are not required for PEDV attachment or internalization

**Figure 3.**
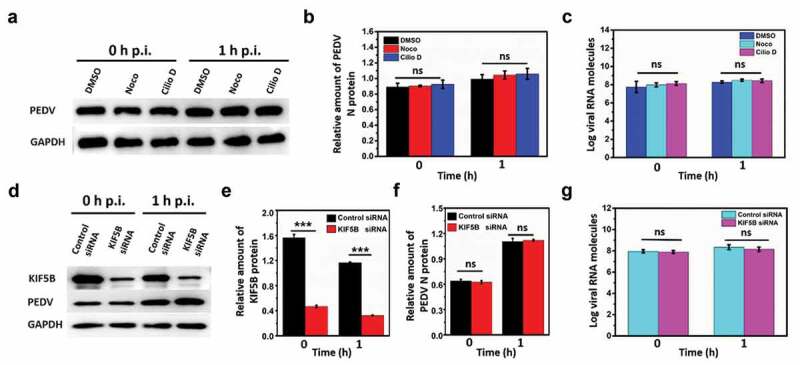
Microtubule, dynein and kinesin-1 are not required for PEDV attachment or internalization. (a) Detection on PEDV N protein and GAPDH using Western blot from the infected PEDV particles in the control DMSO, Noco and Cilio D pretreated Vero cells at 0 h and 1 h PEDV post-infection (p.i.). (b) Quantification on the amount of PEDV N protein according to the amount of GAPDH. (c) Copy numbers of PEDV RNA from the infected PEDV particles in the control DMSO, Noco and Cilio D pretreated Vero cells at 0 h and 1 h PEDV post-infection using RT-PCR. (d) Detection on the proteins of KIF5B, PEDV N protein and GAPDH using Western blot from the infected PEDV particles in the control siRNA and KIF5B siRNA pretreated Vero cells at 0 h and 1 h PEDV post-infection (p.i.). (e) and (f) Quantification on the amount of KIF5B and PEDV N protein according to the amount of GAPDH. (g) Copy numbers of PEDV RNA from infected PEDV particles in the control siRNA and KIF5B siRNA pretreated Vero cells at 0 h and 1 h PEDV post-infection using RT-PCR. Each data point represents mean ± standard deviation from three independent experiments. Statistical analysis of all data was performed using one-way ANOVA (***, P < 0.001). ns: nonsignificant

To verify whether microtubule, dynein, and kinesin-1 affect the attachment and internalization of PEDV particles, Western blot and reverse transcriptase real-time PCR (RT-PCR) were adopted. First, Vero cells, respectively, treated with control DMSO, Noco, and Cilio D were infected with the same amount of PEDV particles at 4°C for 30 min (as 0 h post-infection) for PEDV attachment and then at 37°C for 1 h (as 1 h post-infection) for PEDV internalization. The infected cells were harvested for protein and viral genome extraction. PEDV N protein and GAPDH were detected using Western blot as shown in Figure 3(a), and the amount of PEDV N protein was quantified based on the amount of GAPDH according to the gray value as shown in Figure 3(b), showing that there were no significant changes in the amounts of PEDV N protein in the microtubule and dynein inhibited cells compared to those in the control DMSO treated cells at 0 h or 1 h PEDV post-infection, thus suggesting that microtubule and dynein were not involved in PEDV attachment or internalization. Meanwhile, the copy numbers of PEDV RNA were analyzed using RT-PCR after the viral genome extraction from the control DMSO, Noco, and Cilio D treated cells as shown in Figure 3(c) (the values of RT-PCR results were listed in Table S1). The results show that the copy numbers of PEDV RNA did not change significantly in the microtubule and dynein inhibited cells compared to those in the control DMSO treated cells at 0 h or 1 h PEDV post-infection, which is consistent with protein analysis results, thus further suggesting that microtubule and dynein did not influence PEDV attachment or internalization.

Next, Vero cells, respectively, transfected with control siRNA and KIF5B siRNA were infected with the same amount of PEDV particles at 4°C for 30 min (as 0 h post-infection) for PEDV attachment and then at 37°C for 1 h (as 1 h post-infection) for PEDV internalization. The infected cells were harvested for protein and viral genome extraction. KIF5B, PEDV N protein, and GAPDH were detected using Western blot as shown in Figure 3(d), and the amounts of KIF5B and PEDV N protein were quantified based on the amount of GAPDH according to the gray value as shown in Figure 3(e) and 3(f), respectively. The results show that the amount of KIF5B in the KIF5B siRNA condition changed significantly compared to that in the control condition at 0 h or 1 h PEDV post-infection, while the PEDV N protein did not, illustrating that KIF5B was successfully knocked down in Vero cells, but its knockdown almost had no obvious influence on the PEDV attachment or internalization. Meanwhile, the copy numbers of PEDV RNA were measured using RT-PCR after viral genome extraction from the control siRNA and KIF5B siRNA treated cells as shown in Figure 3(g) (the values of RT-PCR results were listed in Table S1). These results reveal that the copy numbers of PEDV RNA did not change significantly in the KIF5B knockdown cells compared to that in the control siRNA treated cells at 0 h or 1 h PEDV post-infection, which is also consistent with protein analysis results, thus further suggesting that kinesin-1 did not influence PEDV attachment or internalization.

According to the results in Figure 3, it is found that microtubule, dynein, and kinesin-1 are not related to PEDV attachment or internalization, thus proving that the reduction of DiD-labeled PEDV particle fusion and accumulation in the perinuclear region with microtubule and dynein inhibition as well as KIF5B knockdown is mostly induced by the decrease of PEDV intracellular transport. Therefore, observation and analysis on PEDV intracellular transport are helpful to understand how PEDV particles traverse across the cytoplasm to facilitate their infection.

## Five types of PEDV intercellular transport observed by single-virus tracking in live Vero cells

**Figure 4.**
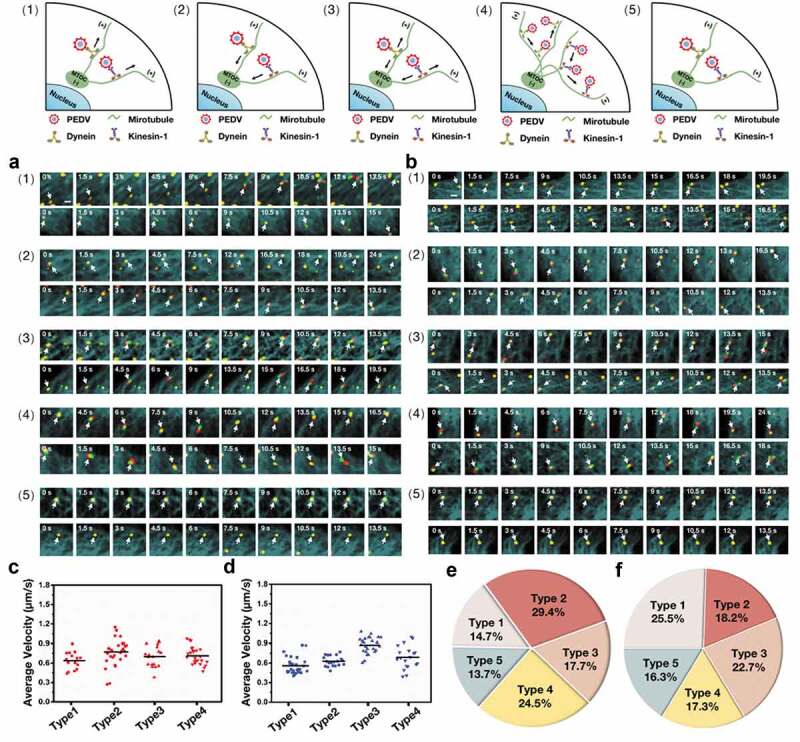
Five types of PEDV intercellular transport observed by single-virus tracking in live Vero cells. (a)-(b) Representative time-lapse fluorescence images of PEDV particles during intercellular transport in live Vero cells transfected with (a) EGFP-MT and mKO2-dynein and (b) EGFP-MT and mKO2-KIF5B in different types: (1) unidirectional movement toward microtubule plus ends, (2) unidirectional movement toward microtubule minus ends, (3) bidirectional movement along the same microtubule, (4) bidirectional movement along different microtubules and (5) motionless state. White arrows indicate PEDV particles. Scale bar, 1 μm. (c) Average velocities corresponding to motion states of PEDV intercellular transport according to (a). (d) Average velocities corresponding to motion states of PEDV intercellular transport according to (b). (e) Proportions corresponding to five types of PEDV intercellular transport according to (a). (f) Proportions corresponding to five types of PEDV intercellular transport according to (b)

To investigate the functions of dynein and kinesin-1 on the PEDV intercellular transport along microtubules, single DiD-labeled PEDV tracking in live Vero cells was performed. 102 DiD-labeled PEDV particles during intercellular transport in live Vero cells transfected with EGFP-MT and mKO2-dynein were observed as shown in Figure 4(a) to analyze the functions of microtubule and dynein (Movie S1), and another 100 DiD-labeled PEDV particles during intercellular transport in live Vero cells but transfected with EGFP-MT and mKO2-KIF5B were also observed as shown in Figure 4(b) to analyze the functions of microtubule and kinesin-1 (Movie S2). In both cases, totally five types of intercellular transport were observed: (1) unidirectional movement toward microtubule plus ends, (2) unidirectional movement toward microtubule minus ends, (3) bidirectional movement along the same microtubule, (4) bidirectional movement along different microtubules and (5) motionless state (their trajectories and velocities are listed in Figure S3). The different average velocities and the proportions of each type are shown in Figure 4(c) and 4(e) for dynein assisted PEDV and Figure 4(d) and 4(f) for kinesin-1 assisted PEDV, respectively.

According to the single-virus tracking results, PEDV particles, microtubules, and dynein/kinesin-1 colocalized during intercellular transport, definitely proving that microtubule, dynein, and kinesin-1 are involved in PEDV intracellular transport.

## Cooperation between dynein and kinesin-1 during PEDV intercellular transport

To further analyze the functions of dynein and kinesin-1 in PEDV intercellular transport, single-virus tracking was still adopted to observe the individual PEDV intracellular transport in live Vero cells but with dynein inhibition and KIF5B knockdown, respectively.

**Figure 5.**
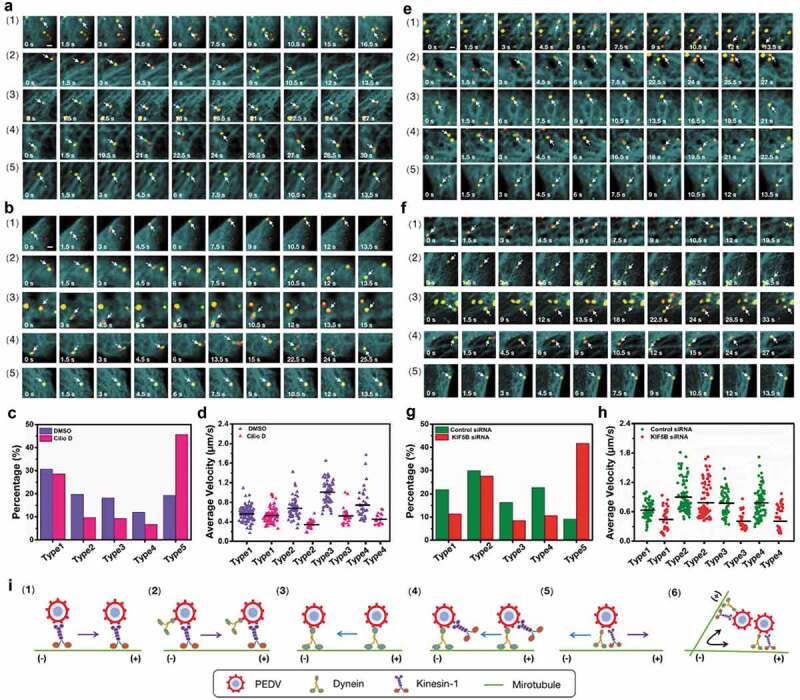
Cooperation between dynein and kinesin-1 during PEDV intercellular transport. (a)-(b) Representative time-lapse fluorescence images of PEDV particles during intercellular transport in live Vero cells transfected with EGFP-MT and mKO2- KIF5B corresponding to the (a) control DMSO treated and (b) dynein inhibited conditions. White arrows indicate PEDV particles. Scale bar, 1 μm. (c) Proportions corresponding to five types of PEDV intercellular transport according to (a) and (b). (d) Average velocities corresponding to motion states of PEDV intercellular transport according to (a) and (b). (e)-(f) Representative time-lapse fluorescence images of PEDV particles during intercellular transport in live Vero cells transfected with EGFP-MT and mKO2-dynein corresponding to (e) control siRNA and (f) KIF5B knockdown conditions. White arrows indicate PEDV particles. Scale bar, 1 μm. (g) Proportions corresponding to five types of PEDV intercellular transport according to (e) and (f). (h) Average velocities corresponding to motion states of PEDV intercellular transport according to (e) and (f). (i) Models corresponding to motion states of PEDV intercellular transport

First, in order to reveal the function of dynein on PEDV intercellular transport, 318 DiD-labeled PEDV particles during intercellular transport were observed in live Vero cells transfected with EGFP-MT and mKO2-KIF5B and treated with control DMSO; but another 300 DiD-labeled PEDV particles during intercellular transport were observed in live Vero cells transfected with EGFP-MT and mKO2-KIF5B but treated with Cilio D for dynein inhibition. Figure 5(a) (Movie S3) and 5(b) (Movie S4) show representative time-lapse fluorescence images corresponding to these two conditions, and in addition, their trajectories and velocities are listed in Figures S4. The colocalization among fluorescence signals from PEDV particles, dynein, and microtubules in Figure 4(a) indicates that PEDV intercellular transport along microtubules was driven by dynein. Moreover, all the five types of PEDV intercellular transport could still be found in these two conditions, but their proportions are quite different as shown in Figure 5(c): when the dynein was inhibited, all the proportions corresponding to Types 1–4 as motion states were reduced but that of motionless state as Type 5 significantly increased compared to those in the control condition. Moreover, the average velocities of PEDV intercellular transport were analyzed as shown in Figure 5(d), illustrating that, in all motion states, the average velocities of PEDV intercellular transport in the dynein inhibition condition decreased compared to those in the control condition. All the results including type proportion and intercellular transport velocity in Figure 5(a)-5(d) further prove that PEDV intercellular transport along microtubules can be driven by dynein.

Next, in order to identify the function of kinesin-1 on PEDV intercellular transport, 320 DiD-labeled PEDV particles during intercellular transport were observed in live Vero cells transfected with EGFP-MT, mKO2-dynein and control siRNA; but another 308 DiD-labeled PEDV particles during intercellular transport were observed in live Vero cells transfected with EGFP-MT, mKO2-dynein and KIF5B siRNA for knockdown of KIF5B. Figure 5(e) (Movie S5) and 5(f) (Movie S6) show representative time-lapse fluorescence images corresponding to these two conditions; and in addition, their trajectories and velocities are listed in Figure S5. The colocalization among fluorescence signals from PEDV particles, kinesin-1 and microtubules in Figure 4(b) indicates that PEDV intercellular transport along microtubules was driven by kinesin-1. Moreover, all the five types of PEDV intercellular transport could still be found in these two conditions, but their proportions were also different as shown in Figure 5(g): when KIF5B was knocked down, all the proportions corresponding to motion states were reduced but that of the motionless state significantly increased compared to those in the control condition. Moreover, the average velocities of PEDV intercellular transport were analyzed as shown in Figure 5(h), also illustrating that in all motion states, the average velocities of PEDV intercellular transport in the KIF5B knockdown condition decreased compared to those in the control condition. All the results including type proportion and intercellular transport velocity in Figure 5(e)-5(h) further prove that PEDV intercellular transport along microtubules can be driven by kinesin-1.

According to the results in Figure 5(a)-5(h), it was suggested that there were two models for Type 1 as shown in Figure 5(i1) and 5(i2): since the inhibition of dynein had little influence on the proportion and the intercellular transport velocity corresponding to Type 1 of kinesin-1 assisted PEDV movement, suggesting that kinesin-1 was mainly responsible for unidirectional movement toward microtubule plus ends according to the model of Figure 5(i1); while knockdown of KIF5B remarkably reduced the proportion and the intercellular transport velocity corresponding to Type 1 of dynein assisted PEDV movement, suggesting that kinesin-1 played a critical role in Type 1 of dynein assisted PEDV movement according to the model of Figure 5(i2). Similarly, there were also two models for Type 2 as shown in Figure 5(i3) and 5(i4): since the knockdown of KIF5B had little influence on the proportion and the intercellular transport velocity corresponding to Type 2 of dynein assisted PEDV movement, suggesting that dynein was mainly responsible for unidirectional movement toward microtubule minus ends according to the model of Figure 5(i3), while inhibition of dynein remarkably reduced the proportion and the intercellular transport velocity corresponding to Type 2 of kinesin-1 assisted PEDV movement, suggesting that dynein played a critical role in Type 2 of kinesin-1 assisted PEDV movement according to the model of Figure 5(i4). In addition, for the bidirectional movements along the same microtubule and different microtubules (Types 3 and 4), it is suggested that dynein and kinesin-1 should cooperatively function on PEDV intracellular transport and with the model of Figure 5(i5) and 5(i6), respectively, since both dynein inhibition and KIF5B knockdown obviously reduced the proportions and the intercellular transport velocities corresponding to Types 3 and 4.

According to the results in Figure 5, it is found that dynein and kinesin-1 mainly transport PEDV to the minus and plus ends of the microtubules, respectively. Meanwhile, dynein and kinesin-1 also can transport PEDV to the opposite ends of the microtubules different from their conventional transport directions and also coordinate the bidirectional movement of PEDV along the same or different microtubules through their cooperation.

## Discussion

**Figure 6.**
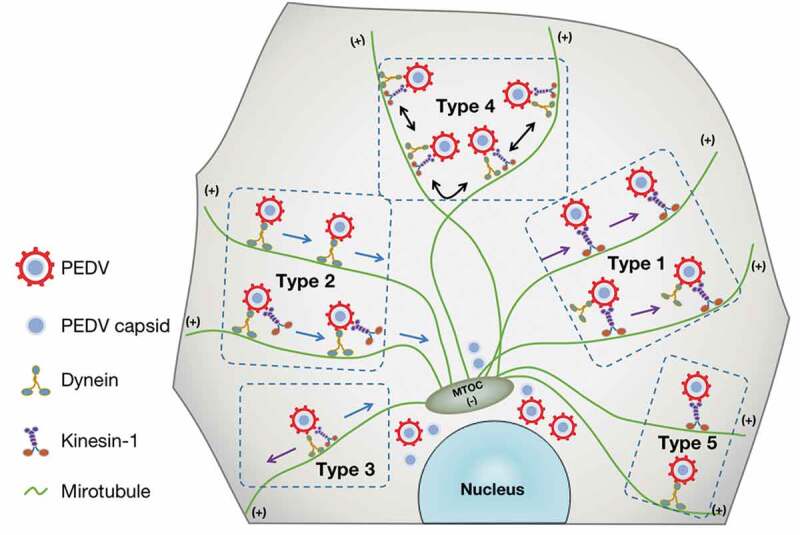
Scheme of the PEDV intercellular transport driven by dynein and/or kinesin-1 along microtubules. After entering the cytoplasm, PEDV particles were driven by dynein and/or kinesin-1 along microtubules in five different types, and these intercellular transports play an important role in PEDV fusion and accumulation in the perinuclear region

Throughout the viral replication cycle, viral proteins, complexes, and particles need to be transported in host cells. However, little is known about how PEDV particles traverse across the cytoplasm to facilitate their infection. Likewise, the intercellular transport on microtubules by dynein and kinesin-1 motors has an important role in the replication and spread of many viruses. Here we have studied the role of microtubule, dynein and kinesin-1 in PEDV infection and have extensively investigated the underlying mechanisms of how microtubule, dynein and kinesin-1 contribute to PEDV infection. We determined that microtubule, dynein, and kinesin-1 are involved in PEDV infection, and can influence PEDV fusion and accumulation in the perinuclear region but cannot affect PEDV attachment or internalization. These results were tempting to speculate that microtubule, dynein, and kinesin-1 are involved in PEDV intercellular transport and thus to influence the PEDV infection. In order to answer this question, we adopted single-virus tracking in this study not only to unveil that the PEDV intercellular transport along microtubules is driven by dynein and kinesin-1 but also to dynamically reveal five different motion types including (1) unidirectional movement toward microtubule plus ends, (2) unidirectional movement toward microtubule minus ends, (3) bidirectional movement along the same microtubule, (4) bidirectional movement along different microtubules and (5) motionless state as shown in Figure 6.

Besides the dynamic observation on PEDV intercellular transport, the specific functions of dynein and kinesin-1 on different PEDV intercellular transport types are also discussed. The unidirectional movement toward microtubule plus ends as Type 1 was observed. In the dynein inhibition condition, it is found that there was little influence on the Type 1 proportion of kinesin-1 assisted PEDV movement, suggesting that kinesin-1 was mainly responsible for unidirectional movement toward microtubule plus ends according to the model in Figure 5 (i1), which is consistent with the previous reports on the function of kinesin-1 [40]. According to the single-virus tracking results, it is also found that dynein could drive PEDV particles toward microtubule plus ends following Type 1 motion, which seems inconsistent with the previous reports that dynein often moves toward microtubule minus ends [40]. However, such PEDV intercellular transport driven by dynein is similar to the anterograde transport of endosomes, peroxisomes, and nuclei assisted with dynein. It has been reported that with the help of Lis 1, dynein can promote the anterograde transport of endosomes, peroxisomes, and nuclei to the axon direction, and Lis1 in this process acts as a dynein promoter in initiating dynein-driven motility [41]. Moreover, there is also evidence that dynein recruitment to the microtubule plus ends depends on Lis1 ortholog (also known as Pac1) [42] and microtubule-binding protein Clip170 (BiK1) [43]. Meanwhile, in our study, we found that after KIF5B knockdown, the Type 1 proportion of dynein assisted PEDV movement significantly reduced, indicating that dynein can also drive PEDV particles to the microtubule plus ends with kinesin-1 together according to the model in Figure 5(i2), which is also consistent with previous reports that dynein can be transported to microtubules plus ends in a kinesin-1 dependent manner in filamentous fungi [41,44–46] and neurons [47,48]. Therefore, it is suggested that dynein may cooperate with the above-described proteins, or even other proteins in cells to transport PEDV toward microtubules plus ends, but still needing further experiments to clarify these possibilities.

The unidirectional movement toward microtubule minus ends as Type 2 was also observed. In the KIF5B knockdown condition, it is also found that there was little influence on the Type 2 proportion of dynein assisted PEDV movement, illustrating that dynein was mainly responsible for unidirectional movement toward microtubule minus ends according to the model in Figure 5(i3), which is consistent with the dynein role in the transport of herpes simplex virus [49] and simian virus 40 [50]. However, according to the PEDV single-virus tracking results, it shows that kinesin-1, which often moves toward microtubule plus ends, could also drive PEDV particles toward microtubule minus ends. One recent study illustrated that the capability to associate with kinesin-1 is actually critical in allowing HIV-1 to achieve its perinuclear location, and also observed that fasciculation and elongation protein zeta-1 (FEZ1) promoted the binding of the viral core to kinesin-1 to achieve net movement toward the nucleus [51], thus proving that kinesin-1 can transport the virus to the nuclear region through the interaction with intracellular proteins. In this study, the Type 2 proportion decrease in dynein inhibition condition indicates that kinesin-1 transport PEDV toward microtubule minus ends through the cooperation with dynein according to the model in Figure 5(i4) or through the cooperation with some other intracellular proteins like FEZ1 which needs to be further studied.

Using single-virus tracking, the bidirectional motion types (Types 3 and 4) in PEDV intercellular transport were observed in our study. It is worth noting that the bidirectional transport movements are also found in Herpes virus [52], adenovirus [53], and HIV [22]. With dynein inhibition or KIF5B knockdown, both Type 3 and 4 proportions significantly reduced, which are consisted with the bidirectional movement reduction by inhibiting either dynein [54] or kinesin [55,56] in extruded squid axoplasm. According to the previous reports that dynein and kinesin-1 can interact directly and specifically [24], it is strongly suggested that they are mutually cooperative through the PEDV bidirectional movements according to the models of Figure 5(i5) and 5(i6). Actually, in cells, there are many cargos such as mitochondria [57], endosomes [58] and secretory vesicles [59] moving bidirectionally and reversing course every few seconds, thus inevitably leading to traffic jams. Bidirectional transport is a normal cellular mechanism enabling the virus to traffic through the crowded cytoplasm: when the virus encounters a roadblock, such as organelle trafficking in the opposing direction along the same microtubule, the virus then undergoes anterograde movement (Type 3) or skips to a neighboring microtubule (Type 4) [27]. Once the virus gets over the obstacle, it continues the retrograde movement toward the nucleus [60].

For the motionless state as Type 5, although these viruses colocalized with dynein or kinesin-1 on the microtubules, they did not move along microtubules but exhibited a permanently stationary state, which is similar to the condition that motor changes from a transient pause to a permanent suspension when it encounters obstacles [61]. The reason for this phenomenon is probably that the motor will detach and reattach the nearby protofilament when encountering the obstacle, but this mechanism is not robust enough to effectively get over the obstacle, and thus exhibits a permanent pause.

In our study, we only clarified that microtubule, dynein, and kinesin-1 were involved in PEDV accumulation in the perinuclear region, but why microtubule, dynein, and kinesin-1 affect PEDV accumulation to the perinuclear region is still unknown. However, based on the previous reports [12,34,62–64] as well as the accumulation of PEDV in the perinuclear region observed in our study, we have several speculations. It was reported that the tubulins could interact with the last 39 amino acid stretches of the spike (S) protein cytoplasmic tail of Alphacoronaviruses, and such interaction affected the distribution of S proteins in the perinuclear region [62]. Therefore, it is speculated that PEDV accumulation in the perinuclear region might be related to the interaction between PEDV S protein and the microtubule. Additionally, it was revealed that the internalized influenza virus experienced an intermittent active transport involving both plus- and minus-end-directed motor proteins on the microtubule in the perinuclear region through an early endosome-late endosome pathway, and its initial acidification step also occurred in the perinuclear region [34]. Furthermore, it was illustrated that the internalized PEDV was also transported to the early and late endosomes [12], and in our study, the microtubule, dynein, and kinesin-1 were involved in the PEDV intercellular transport, so it is speculated that PEDV accumulation in the perinuclear region by microtubule, dynein, and kinesin-1 might promote the replication process of PEDV through acidification. However, because the replication mechanism of the influenza virus is different from that of PEDV, that is, influenza virus replicates in the nucleus [63], while PEDV replicates in the cytoplasm [64], the reason for PEDV accumulation in the perinuclear region is another meaningful work and should be further studied.

In conclusion, we dynamically observed five different motion types of PEDV intercellular transport and analyzed the functions of microtubule, dynein, and kinesin-1 on this process. The results in this study not only provide an in-depth understanding of the mechanism of PEDV infection but also facilitate the development of effective drugs and new vaccines for PEDV infection. In addition, it will also be interesting to determine whether the microtubule, dynein, and kinesin-1 are also acting similar roles in other envelope virus infection.

## Supplementary Material

Supplemental Material
