# Supplementary material for: Dynamic Dissection of Dynein and Kinesin-1 Cooperatively Mediated Intercellular Transport of Porcine Epidemic Diarrhea Coronavirus along Microtubule Using Single Virus Tracking
Source: Virulence. 2021 Feb 4;12(1):615–29. doi: 10.1080/21505594.2021.1878748 (PMC7872075; doi:10.1080/21505594.2021.1878748)
Supplement: Supplemental Material [file KVIR_A_1878748_SM8304.docx]

**Supplementary Information**

**Dynamic Dissection of Dynein and Kinesin-1 Cooperatively Mediated Intercellular Transport of Porcine Epidemic Diarrhea Coronavirus along Microtubule Using Single Virus Tracking**

Wei Hou^1^, Wenjie Kang^1^, Yangyang Li^1^, Yanke Shan^1^, Shouyu Wang^1,2^ and Fei Liu^1*^

^1^Joint International Research Laboratory of Animal Health and Food Safety of Ministry of Education & Single Molecule Nanometry Laboratory (Sinmolab), Nanjing Agricultural University, Nanjing, Jiangsu, 210095, China

^2^Computational Optics Laboratory, Jiangnan University, Wuxi, Jiangsu 214122, China

*Corresponding author: feiliu24@njau.edu.cn (F.L.)


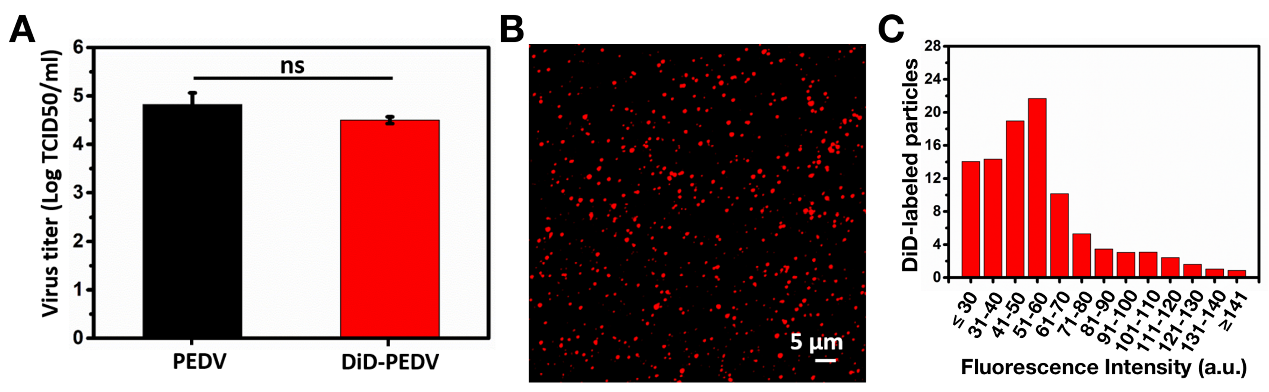


**Figure S1. Characterization on DiD-labeled PEDVs.** (A) Virus titers of unlabeled and DiD-labeled PEDV particles, showing that DiD did not significantly influence the PEDV infectivity assessed by TCID_50_. Each result was obtained from 9 independent experiments. ns: nonsignificant. (B) Representative fluorescence image of DiD-labeled PEDVs. Scale bar, 5 μm. (C) Intensity histogram of 7009 individual DiD-labeled PEDVs, suggesting that DiD was almost uniformly labeled on the PEDVs. Particles with fluorescence intensity under 70 a.u. were selected for image analysis, since these most probably resemble single virus particles. Each data point represents mean ± standard deviation. a.u.: arbitrary units.

**
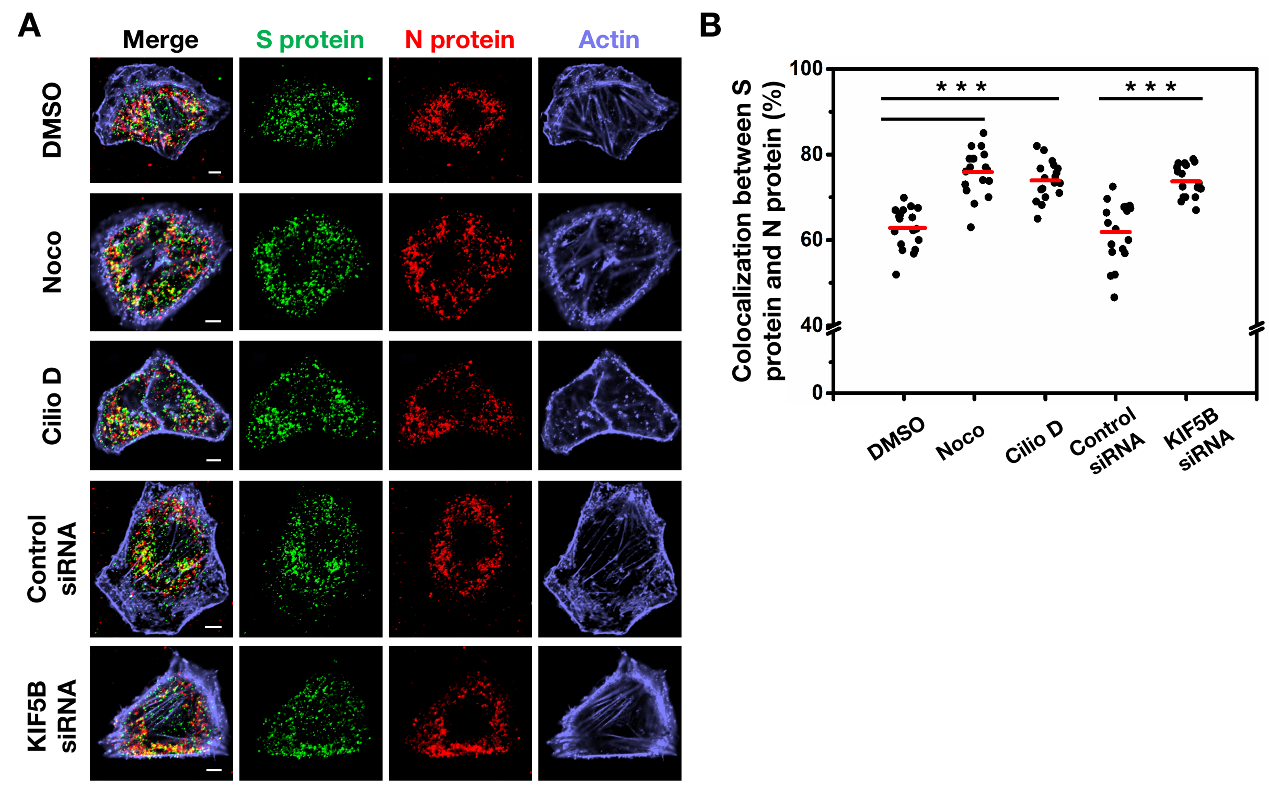
**

**Figure S2. Microtubule, dynein and kinesin-1 affect PEDV fusion.** (A) The colocalization between PEDV S protein and N protein in Vero cells first respectively treated with control DMSO, Noco, Cilio D, control siRNA and KIF5B siRNA, then infected with PEDV particles for 1 h and finally fixed. The infected Vero cells were incubated with the PEDV S protein polyclonal antibody and N protein monoclonal antibody at 37 °C for 1 hour, respectively, and then incubated with the Dylight 549- and Fluor 647-conjugated secondary antibody at 37 °C for 1 hour, respectively. Actin was stained with AbFluor 488-conjugated phalloidin. Scale bar, 5 μm. (B) Statistical analysis on the colocalization between PEDV S protein and N protein from (A) (n=18 cells).


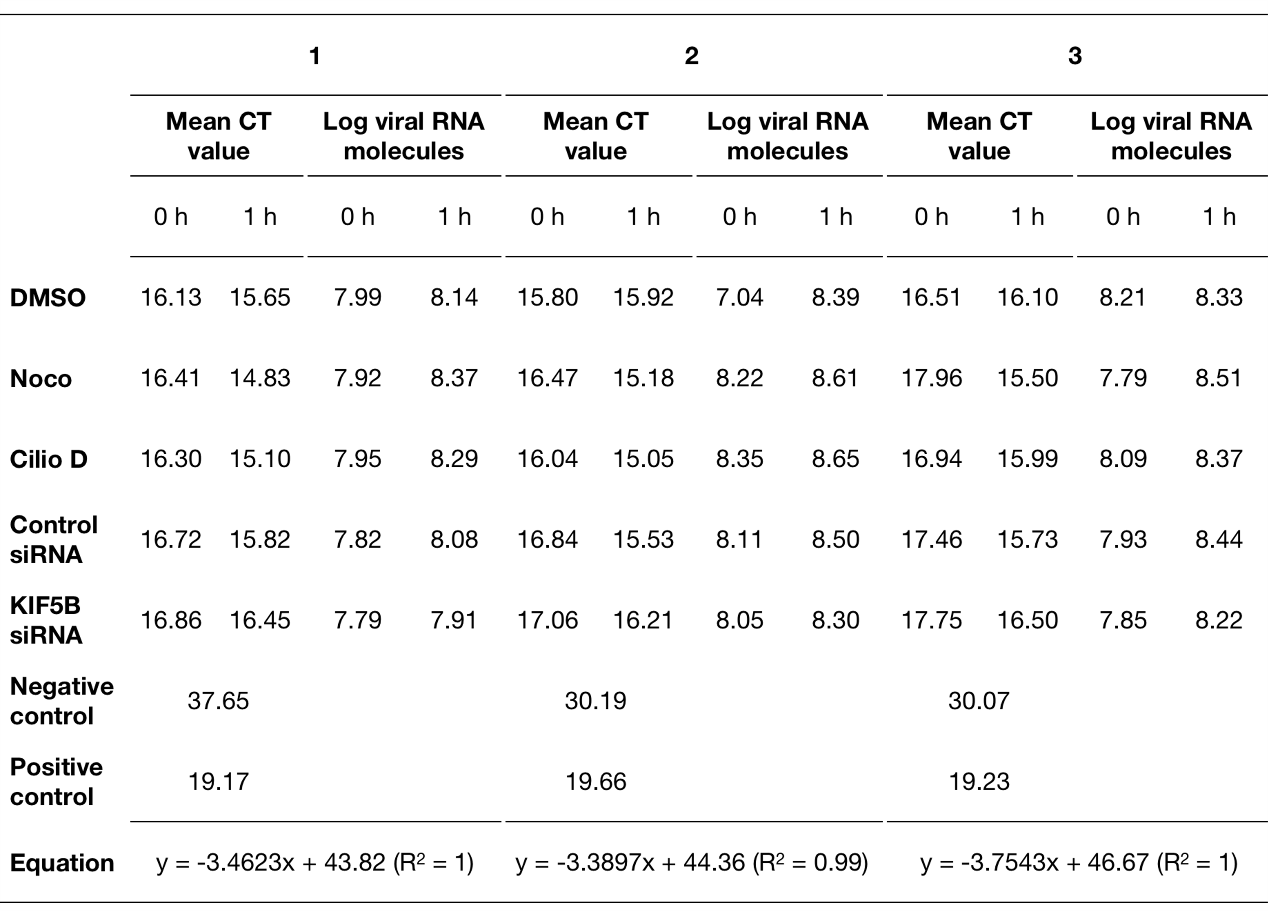


**Table S1. RT-PCR results.**

**
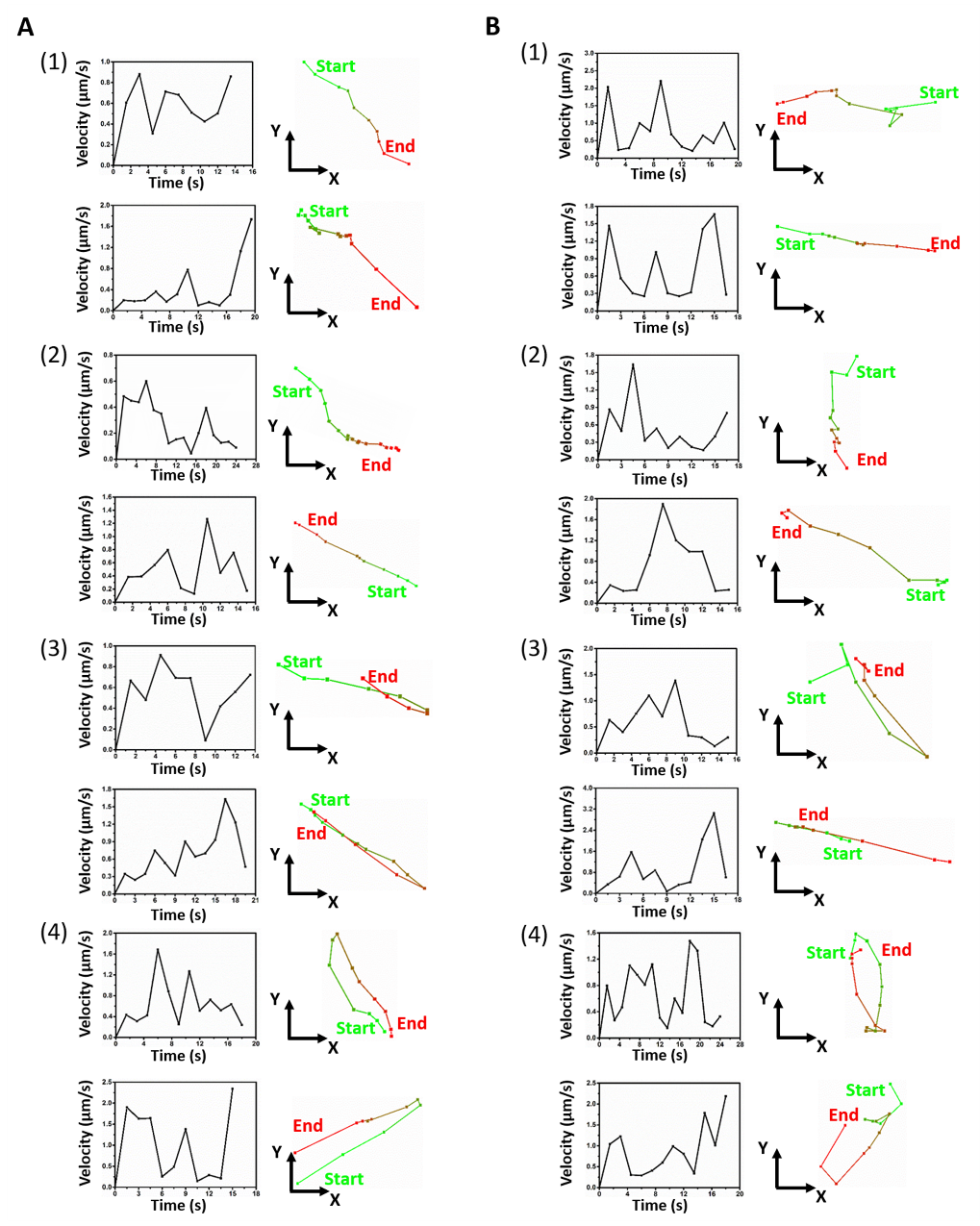
**

**Figure S3. Velocities and trajectories corresponding to motion states of PEDV intercellular transport observed by single virus tracking in live Vero cells.** (A) Vero cells were transfected with EGFP-MT and mKO2-dynein. (B) Vero cells were transfected with EGFP-MT and mKO2-KIF5B.

**
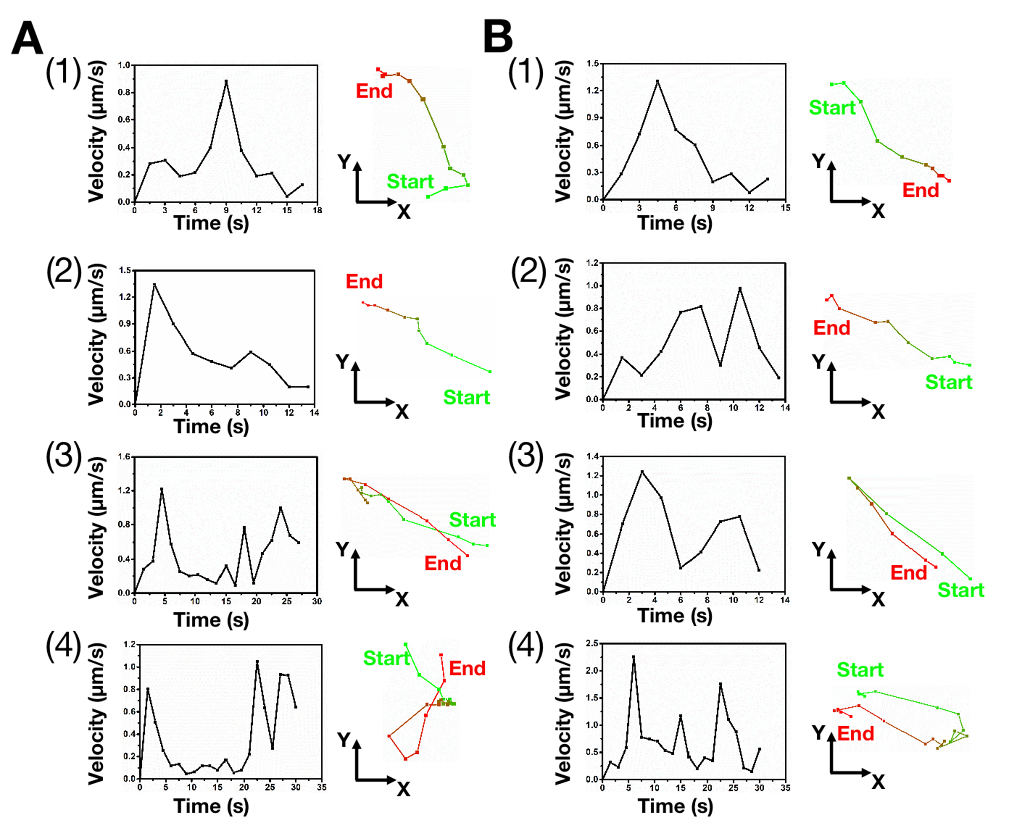
**

**Figure S4. Velocities and trajectories corresponding to motion states of PEDV intercellular transport observed by single virus tracking in live Vero cells.** Vero cells transfected with EGFP-MT and mKO2-KIF5B and treated with (A) control DMSO and (B) Cilio D.


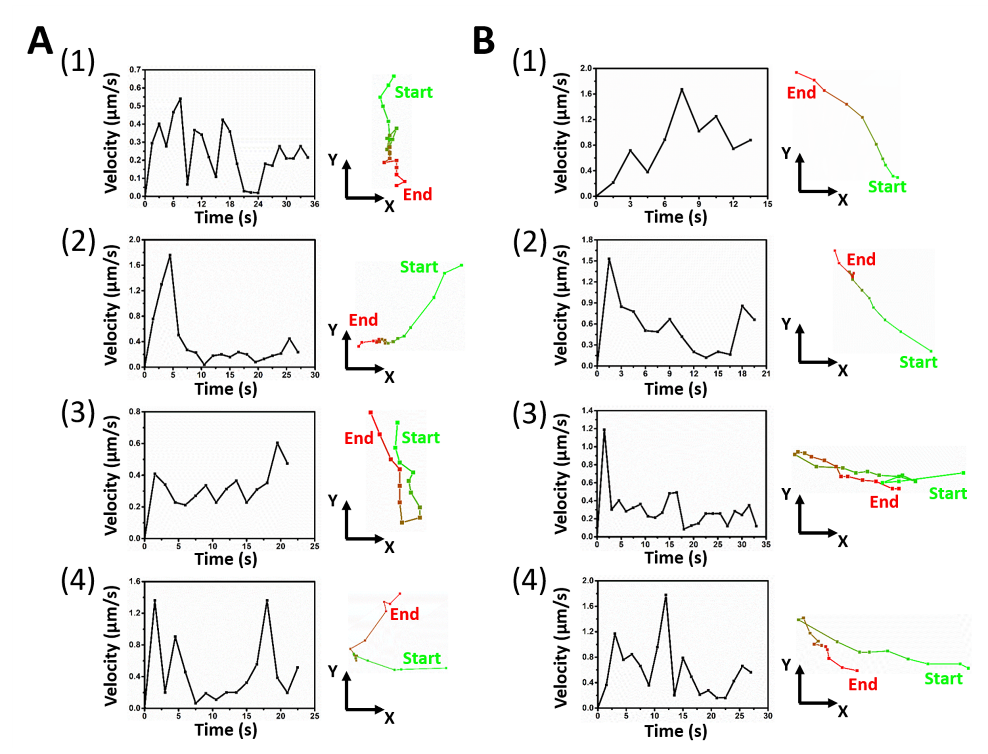


**Figure S5. Velocities and trajectories corresponding to motion states of PEDV intercellular transport observed by single virus tracking in live Vero cells.** Vero cells transfected with EGFP-MT and mKO2-dynein and treated with (A) control siRNA and (B) KIF5B siRNA.

**Supporting Movies**

Movie S1: Time-lapse images of type 1-5 movements of dynein driven PEDV along microtubule.

Movie S2: Time-lapse images of type 1-5 movements of kinesin-1 driven PEDV along microtubule.

Movie S3: Time-lapse images of type 1-5 movements of kinesin-1 driven PEDV along microtubule in control DMSO condition.

Movie S4: Time-lapse images of type 1-5 movements of kinesin-1 driven PEDV along microtubule in Cilio D condition.

Movie S5: Time-lapse images of type 1-5 movements of dynein driven PEDV along microtubule in control siRNA condition.

Movie S6: Time-lapse images of type 1-5 movements of dynein driven PEDV along microtubule in KIF5B siRNA condition.
